# Is Hahnemann Obsolete?

**Published:** 1881-03

**Authors:** B. H. Cheney

**Affiliations:** New Haven, Conn.


### IS HAHNEMANN OBSOLETE?

BY B. H. CHENEY, M.D., NEW HAVEN, CONN.

THE following extracts, headed "Reynolds," are from the introduction to "Reynoldd's System of Medicine," which is an acknowledged exponent of the very latest facts and opinions in "regular" medicine. The author of the introduction, who is also the editor of the whole work, is Prof. J. Russell Reynolds, of University College, London. The quotations from Hahnemann are from the "Organon" as indicated by sections, Wesselhoeft's translation.

| REYNOLDS, A.D., 1878. | HAHNEMANN, A.D., 1808 |
|---|---|
| "So long as 'disease' is thought of as a something —it matters not what—distinct from the 'phenomena,' or 'symptoms,' by which it makes itself known so long are we in danger of mistaking its real meaning, and of overlooking | "§ 18. It is then unquestionably true that, besides the totality of symptoms, it is impossible to discover any other manifestation by which diseases could express their need of relief. Hence it undeniably follows that the totality of |

those true guides toward the removal or alleviation of its evil, an end to which all medical science ultimately points." (Vol. 1, p. 24, Am. edit.)

symptoms observed in each individual case of disease can be the only indication to guide us in the selection of a remedy." Cf. also §§ 12, 13, and 14.

Dr. Reynolds defines disease as, "a change of structure or function, or both," (p. 24); Hahnemann in § 12 speaks of it as an "aberration from healthy vital function," which of course implies a greater or less change in structure, temporary or permanent. The latter seems to us the more comprehensive definition, although the more concise, for it carries with it that idea of the continuance of tissue or cell life, but in an abnormal state, which we consider essential to a right understanding of the scope of pathology.

As regards the vexed question of symptomatology and pathology, the usual definition of the latter is too narrow. For, as by physiology we understand the science of life expressed in natural (healthy) function, so ought pathology to convey the idea of life expressed in unnatural (deranged) function, but *life* nevertheless. Hence the *symptoms* caused by such deranged function are really as important parts of the pathology of the disease as they are of the pathogenesis of the corresponding remedy. From this follows, of course, the necessity of individualization in each case. The changes in structure consequent upon deranged function are results which belong to morbid histology and anatomy, but these are not all there is of pathology.

Again, although "subjective symptoms" are not by any means synonymous with "mental symptom," still the following extracts will bear quotation together.

REYNOLDS.

"We have to deal with man as a whole; and to ignore or undervalue what he tells us

HAHNEMANN.

"§ 211. The state of the patient's mind and temperament * * * is a distinct and of his ideas, emotions, or sensations, because they may be termed 'subjective symptoms' and be held to be, therefore, unreliable, would be to shut out from ourselves that which —egotistic and fearful, prejudiced and ignorant as man may be—yet forms an integral part of his life, and therefore of his disease." (Vol. 1, p. 25, Am. edit.)

peculiar symptom that should least of all escape the accurate observation of the physician.

"§ 212. The effect upon the state of mind and disposition is the principal feature of all diseases, and seems to have been specially ordained by the Creator of all healing powers * * *" Cf. also § 213.

In the last section quoted from the "Organon," the therapeutic parts are purposely omitted to show the correspondence in theory between Hahnemann and Reynolds. But it is to be remembered that Hahnemann did not deduce his therapeutics from his theories. On the contrary, having found by *induction* the law of cure, he framed his theories to accord with it. Hence, the last clause of § 212 was added in proof of the statement in the first clause, quoted above, and should be preceded in the translation by "for," or "since," original (*indem*), as follows; "For there is not a single potent medicinial substance that does not possess the power of altering perceptibly the mental condition and mood of a healthy person, etc."

Hahnemann, having established his therapeutic facts by induction, they can be in no way affected by the fall of any, or all, of his theories. How different with all other so-called "systems" of therapeutics, let their ruins throughout the history of medicine, *passim*, attest. It is safe to assert that neither Dr. Reynolds, nor any one else, no matter how correct his theories, can ever, by *deduction* from them, arrive at any system of therapeutics having the character of *law*, or which can be anything else than uncertain, unstable, and causing constant disappointment. It is beginning at the wrong end. Perhaps in another seventy years this may be seen also.

In the mean time, something is already gained, and, as we look over the medical world, we may say with Galileo, "*E pur si muove!*"

[As an additional proof of the correctness of Hahnemann's teachings, and to show that he is not yet obsolete, we quote the following report on the contagiousness of syphilis. When Hahnemann (see "Chronic Diseases," vol. 1, p. 56) asserted that contagion could be spread by mere touch of clothing or person, etc., the savants of that day ridiculed the idea; now, many years later, they adopt it.

THE AMERICAN PUBLIC HEALTH ASSOCIATION AND THE PREVENTION OF VENEREAL DISEASES.—A committee was appointed by the American Public Health Association, a year ago, to investigate and report upon the subject of venereal diseases and the means of prevening their spread. This committee made its report through Dr. Albert Gihon, U.S.A., at the recent meeting of the association in New Orleans. The committee asserted their belief in the efficacy of regulating prostitution, but they would not recommend the measure at present. They said that as a safeguard and warning everybody should know the following facts—that venereal diseases are communicable:

1. By the blankets, etc., of a sleeping-car, and the sheets, towels, and napkins of hotels and restaurants

2. By the dresses, costumes, etc., rented for fancy balls.

3. By the chipped edge of the coffee-cup; and by the half-cleaned knives, forks, and spoons of restaurants and hotels.

4. By the drinking-vessels in a railway car or station.

5. By barbers' utensils—brush and comb; by hatters' measure, or by a borrowed or sample hat.

6· By surgeons' or dentists' instruments; by the vaccinator or lancet.

7. By toys sold to children by vendors who have been handling them with poisoned lips or fingers.

8. By the broom or dust-brush handled by the housemaid, or by the spoon fouled by the mouth of the cook.

9. By playing-cards and visiting-cards which have been used by syphilitics; by car-tickets and paper money whlch circulates in a city where there are many syphilitics.

10. By the pipe, cane, or glove loaned to a friend.

11. By the grasp of a friend's hand or the kiss of an accepted lover.

In view of this alarming state of things the committee reported the following resolution, which after some debate was adopted by the Association:

*Resolved*, That the American Public Health Association earnestly recommend the municipal and state boards of health to urge upon the legislative bodies of this country the enactment of a law constituting it a criminal offence to knowingly communicate, by any direct or indirect means, a contagious disease; such as small-pox, scarlet-fever, or venereal disease; and giving to said boards of health and to the state and municipal health officers under their control the same power in the prevention, detention, and suppression and gratuitous treatment of venereal affections which they now possess in the case of small-pox and other contagious diseases.—Ed.].

